# Associations between residential greenness, land cover and risk of celiac disease in genetically at‐risk children: Celiac Prediction in Skåne study

**DOI:** 10.1002/jpn3.70440

**Published:** 2026-04-22

**Authors:** Michaela Boström, Marja I. Roslund, Lauri Häme, Juulia Manninen, Matthieu Molinier, Aki Sinkkonen, Daniel Agardh

**Affiliations:** ^1^ Department of Clinical Science Lund University Malmö Sweden; ^2^ Natural Resources Institute Finland (Luke) Helsinki Finland; ^3^ Terramonitor Oy Helsinki Finland; ^4^ Ecosystems and Environment Research Programme Faculty of Biological and Environmental Sciences University of Helsinki Helsinki Finland; ^5^ VTT Technical Research Centre of Finland Ltd Helsinki Finland

**Keywords:** autoimmunity, environmental exposure, leaf area index, normalized difference vegetation index, remote sensing

## Abstract

**Objectives:**

Our aim was to study the association between residential land cover and greenness during childhood and risk of developing celiac disease (CeD).

**Methods:**

The Celiac Prediction in Skåne study prospectively followed 5969 human leukocyte antigen (HLA)‐genotyped children born 2001–2004 who were screened for CeD at ages 3, 9, and 15 years in Skåne, Sweden. Among these, 138 (2.3%) children only in the HLA at‐risk group were identified and diagnosed with CeD through screening. Children diagnosed with CeD outside the screening program were excluded. For the present study, 2535 children at HLA‐risk were included. Residential addresses at birth and screening time points were transformed into spatial coordinates. Coordination of Information on the Environment Land Cover data was collected the corresponding years. Normalized Difference Vegetation Index and Leaf Area Index (LAI) were calculated for the summer months.

**Results:**

Higher residential LAI, an indicator of forest and agricultural land cover within a 500‐meter buffer, was associated with increased odds of CeD in adjusted logistic regression models at age 3 (OR 1.52, 95% CI: 1.14–1.99) and age 9 (OR 1.62, 95% CI: 1.13–2.27). After false discovery rate adjustment, several associations with LAI remained statistically significant, whereas no land cover classes were associated with CeD.

**Conclusions:**

Residential greenness was associated with increased CeD risk in the Skåne province. This exploratory observational finding warrants replication in independent populations before conclusions can be drawn regarding potential environmental drivers in the etiology of CeD.

## Abstract (highlights)

**What is Known:**

- Celiac disease (CeD) develops in genetically predisposed individuals, and several environmental factors have been proposed; however, evidence regarding the impact of broader environmental and residential exposures remains limited.
- Associations between residential greenness and other immune‐mediated diseases have been reported previously.

**What is New:**

- To our knowledge, this is the first study to examine the association between residential greenness, land cover, and CeD.
- Higher residential Leaf Area Index, indicating more greenness, was associated with an increased risk of CeD in age‐specific analyses, with some associations remaining statistically significant after correction for multiple testing.

## 1 INTRODUCTION

Incidence of several autoimmune diseases, including celiac disease (CeD) is increasing,^1^ suggesting that environmental exposures beyond genetic susceptibility contribute to disease development. One proposed mechanism is reduced contact with natural biodiversity, which may impair immune regulation.^2^ Alterations in the gut microbiota have also been implicated in CeD pathogenesis and progression.^3^, ^4^ As early‐life environments shape microbial exposure and immune maturation, childhood residential environments may play an important role in disease onset.

Residential environmental characteristics can be quantified using remote sensing‐derived measures, including Coordination of Information on the Environment (CORINE) Land Cover data and vegetation indices such as Leaf Area Index (LAI) and Normalized Difference Vegetation Index (NDVI).

Associations between land cover, residential greenness and immune‐mediated diseases have been reported.^5^, ^6^, ^7^, ^8^, ^9^ For example, living in agricultural environments has been inversely associated with the risk of type 1 diabetes,^6^ and higher residential greenness has been linked to lower risks of inflammatory bowel disease and atopy.^7^, ^9^ Conversely, land cover characterized by soil sealing and urbanization has been associated with increased risks of type 1 diabetes and asthma.^5^, ^6^ However, findings are not consistent, as greater residential greenness has also been associated with increased odds of childhood wheezing, asthma, and allergic rhinitis.^8^

The aim of the present study was to investigate whether childhood residential environmental characteristics, assessed using satellite‐derived CORINE Land Cover data and greenness indices, were associated with CeD in Skåne, Sweden. We hypothesized that higher residential greenness (LAI and NDVI) and green land cover types would be inversely associated with CeD, whereas land cover characterized by soil sealing would be associated with increased risk of CeD.

## 2 METHODS

### 2.1 Ethics statement

The Celiac Prediction in Skåne (CiPiS) studies were reviewed and approved by the Ethics Committee of the Medical Faculty, Lund University (Dnr Lu 878‐02, Dnr 2010/170, Dnr 2011/335, Dnr 2016/410, Dnr 2021‐04470). Written informed consent to participate in this study was provided by the participants' legal guardian/next of kin.

### 2.2 Study population

Study participants were included from the prospective cohort study CiPiS, as previously described.^10^, ^11^, ^12^ In CiPiS, children born between 2001 and 2004 were human leukocyte antigen (HLA) genotyped at birth and invited to participate in screening for CeD using transglutaminase autoantibodies (tTGA) analyzed at ages 3, 9, and 15 years, respectively. Intestinal biopsies were performed in persistently tTGA‐positive children to confirm diagnosis of CeD. Among 5969 screened children, 2778 (46.5%) carried HLA risk alleles (DRB1*02 and/or *0302). A total of 138 children were diagnosed with CeD through the CiPiS screening program, all within the HLA‐risk group. Among these, 56 of 3459 (1.6%) children were diagnosed at age 3,^10^ 72 of 4078 (1.8%) at age 9,^11^ and 10 of 2374 (0.4%) at age 15 years,^12^ respectively.

Children who were diagnosed with CeD outside the screening program, including those identified through regular clinical care or self‐report at follow‐up visits, were excluded from the screening and from the present analyses. In total, 47 additional CeD cases, diagnosed outside of the CiPiS screening, were registered among children born between 2001 and 2004 in Skåne.

For the present study, only the children at HLA‐risk were included. Their addresses of residence according to the population register at birth and at the time of the invitation to the screening (at 3, 9, and 15 years of age) were identified retrospectively. The address of the mother was used if not stated being registered at the father. These addresses were transformed into coordinates using Nominatim geocoding (nominatim.org). In case the program was unsuccessful the coordinates could in some cases be found manually using Google Maps. Coordinates were available from 2535/2778 (91.3%) children, and 1230 (48.5%) of those were females.

Coordinates were retrieved from residential addresses of 1774 children at birth, 2403 children at age 3 years, 1927 children at age 9 years, and 961 children at age 15 years, respectively. Known change of residential address during the follow‐up occurred at least once in 1031/2535 (40.7%) children, of whom 578 (56.1%) moved between birth and age 3 years.

Each analysis was cross‐sectional at ages 3, 9, and 15 years, using the residential address recorded at that time point. Because residential history between the screening time points was unavailable, the address at each age was considered the relevant exposure location for that specific analysis.

Children diagnosed with CeD at a previous screening round were not included in subsequent screenings. Consequently, their residential addresses were not reassessed, and they were excluded from analyses at later ages; children diagnosed at age 3 were excluded from analyses at ages 9 and 15, and those diagnosed at age 9 were excluded from analyses at age 15.

Socio‐economic factors were collected from questionnaires received from the visits at 2 months age and at age 2 years, respectively, which included data on parental smoking habits during or after pregnancy (yes/no), whether the mother was working away from home during pregnancy (yes = any time during pregnancy/no), parental educational level (below university/university), and whether the mother or father were born in Sweden (yes/no) (Table S1).

### 2.3 CORINE Land Cover category classification

Land cover surrounding the residential addresses of the study participants was characterized using the preclassified CORINE Land Cover raster data at 100 × 100 m spatial resolution, provided by the Copernicus Land Monitoring Service.^13^ CORINE Land Cover data are available for 39 European countries and are organized hierarchically into three levels: level 1 (five broad classes), level 2 (15 thematic subclasses), and level 3 (44 detailed land cover categories), of which 35 are represented in Sweden.

The CORINE Land Cover data are produced by national mapping agencies following standardized criteria defined by the European Environment Agency. It is based on visual interpretation of high‐resolution satellite imagery, complemented by national in situ data, satellite image processing, and geographic information system integration.

For each participant, land cover exposure was assessed at the corresponding study follow‐up year (2000, 2006, 2012, and 2018). Pixel‐based raster data were aggregated to calculate the proportional coverage of each land cover category within circular buffer zones of 500 and 1500 m radius around the residential coordinates. These proportional measures were subsequently used as continuous exposure variables in the statistical analyses.

All land cover categories present in the study region were included in the dataset and considered in the statistical procedures. To improve interpretability, land cover categories with very low proportional coverage are summarized descriptively in Tables S2–S5, while they are not presented in the regression tables (Tables S6–S9).

### 2.4 NDVI and LAI

The NDVI is a widely‐used metric for quantifying vegetation greenness and the density of vegetation using remote sensing data.^14^ NDVI is effective to distinguish dense forest, nonforest, and agricultural fields, and to determine evergreen forest versus seasonal forest types.^15^ LAI is a vegetation structural variable, defined as the total one‐sided leaf area per unit ground area. It quantifies the amount of leaf material in a canopy, and is therefore a strong indicator of volumetric biomass within an ecosystem, as well as a critical variable scaling photosynthesis, respiration, and evapotranspiration.^16^

NDVI and LAI were obtained from the Moderate Resolution Imaging Spectroradiometer (MODIS) sensor onboard Terra satellite 16‐day composites at 250 m and 500 m spatial resolutions, respectively (MOD13Q1 and MOD15A2H products). The data were downloaded from NASA's Earth Observing System Data and Information System (EOSDIS) for 19 summers (June–July–August), covering the period 2001–2019. For each year and each participant, mean summer NDVI and LAI values were calculated within 500 and 1500 m buffers around the residential address. These annual summer mean values were used in the statistical analyses.

### 2.5 Statistical analysis

Association between CeD and land cover metrics (LAI, NDVI, and land cover proportions) were assessed using logistic regression models, implemented as generalized linear models with a binomial family and logit link using *glm* function in R (version 4.5.0).^17^ The cases consisted of the children ever diagnosed with CeD in CiPiS, and the controls those who did not.

Because the study was exploratory, no single exposure metric was specified a priori; LAI, NDVI, and CORINE Land Cover categories were all evaluated as complementary indicators of the residential environment.

Three prespecified models were fitted for each exposure and time point. Crude model, *Model 1*, included the exposure only. *Model 2* included sex, season of birth, maternal age at delivery, and maternal smoking during pregnancy, selected a priori based on established or plausible associations with CeD and early life environmental exposures. *Model 3* additionally included maternal smoking after pregnancy, paternal smoking, maternal employment outside the home during pregnancy, parental educational level, and parental country of birth, to account for broader socioeconomic and familial factors.

Sensitivity analyses excluding parental smoking after pregnancy were conducted due to substantial missingness and were used to assess robustness rather than to redefine the primary adjustment strategy. Results were comparable between models, supporting that the main findings were not driven by data in the smoking variables.

We assessed effect modification by including single interaction terms between LAI and each potential modifier in logistic regression. Stratum‐specific odds ratios along with Wald *p*‐values were derived from the interaction model, and *P* interaction was obtained from a likelihood‐ratio test comparing models with and without the interaction. Maternal age was categorized in ages <25, 25–34, and ≥35 years (Table S10).

Spearman rank correlation were used to examine associations between LAI and CLR‐transformed CORINE Land Cover classes within the 1500‐m buffer zone. Rare land cover classes, defined as those with nonzero coverage in fewer than 5% of participants, were excluded, and analyses were conducted descriptively by follow‐up age.

Covariates were compared with a t‐test or chi‐square between cases and controls (Table S1). A significance level of *p* < 0.05 was used. To control for multiple testing, *p*‐values were adjusted using the Benjamini–Hochberg false discovery rate (FDR) procedure.

## 3 RESULTS

### 3.1 LAI was directly associated with CeD risk at ages 3 and 9 years

**Figure 1:**
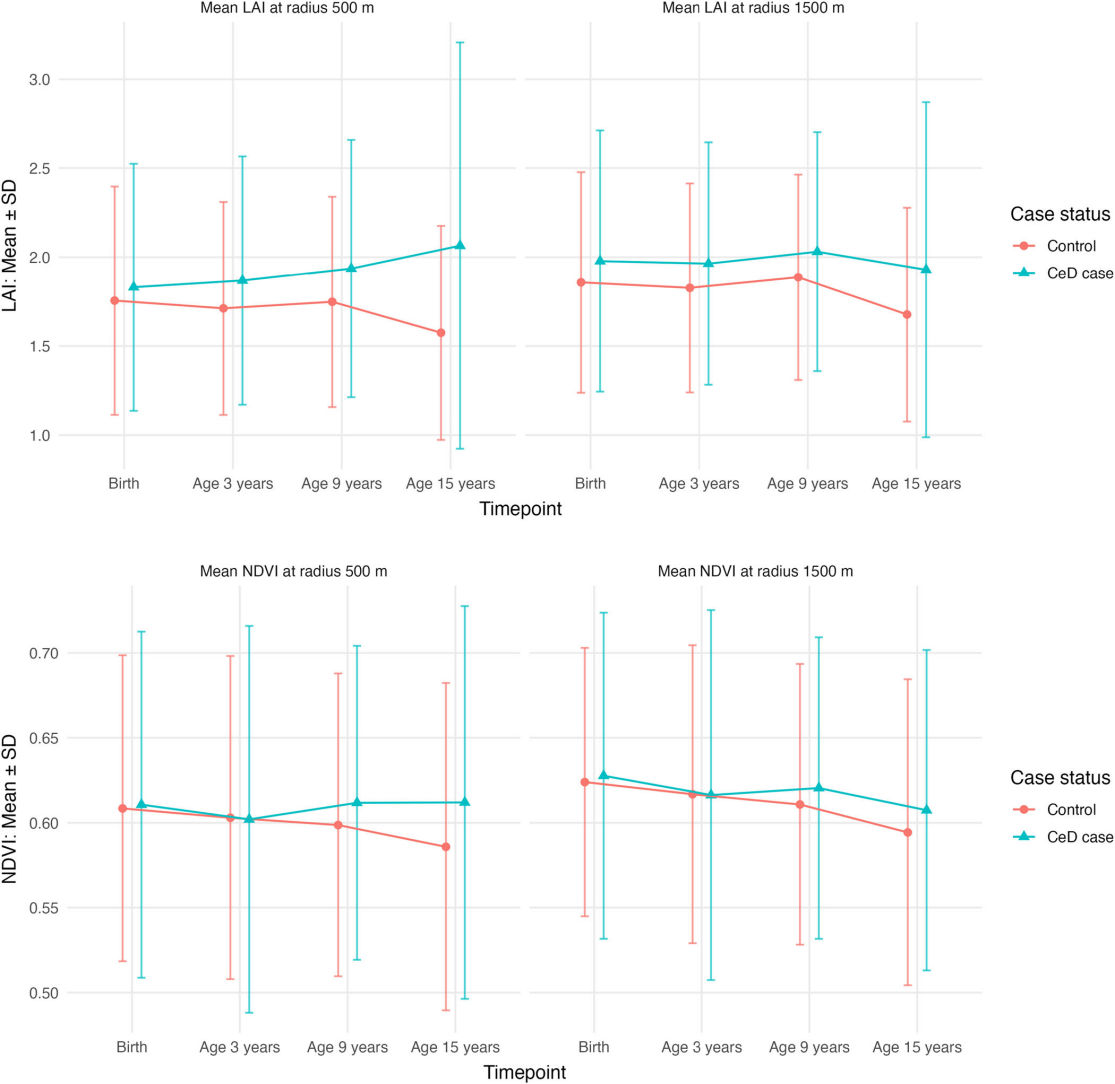
LAI and NDVI over time by CeD status. Displayed are buffer zones around home of the participants in the CiPiS study, of 500 and 1500 m. CeD, celiac disease; CiPiS, Celiac Prediction in Skåne; LAI, Leaf Area Index; NDVI, Normalized Difference Vegetation Index.

Mean exposure to LAI and NDVI for cases and controls at birth and at follow‐up ages 3, 9, and 15 years are summarized in Figure 1 and Table S11.

Logistic regression analyses revealed a positive association between LAI and CeD risk at ages 3 and 9 years.

At age 3, the association remained statistically significant after FDR correction in the crude model (Model 1) and after adjustment for sex, season of birth, maternal age at delivery, and maternal smoking during pregnancy (Model 2) at both the 500 and 1500 m radii. In the fully adjusted model (Model 3), the association did not remain statistically significant when accounting for multiple testing using FDR correction (Table 1). No clear association between NDVI and CeD risk was observed.

**Table 1:** Association of residential greenness at age 3 years and CeD in follow‐up.

Exposure	Model	OR (95% CI)	Cases	Controls	*p*‐Value	*p*‐Adj	Forest
LAI 500 m	1	1.45 (1.10–1.90)	116	1928	**0.01**	**0.02**	│ ──────●──────
	2	1.52 (1.14–1.99)	114	1907	**0.003**	**0.01**	│ ───────●──────
	3	1.48 (1.04–2.06)	79	1257	**0.02**	0.09	│────────●────────
LAI 1500 m	1	1.43 (1.08–1.87)	128	2191	**0.01**	**0.02**	│ ──────●──────
	2	1.51 (1.13–1.99)	126	2168	**0.004**	**0.01**	│ ───────●──────
	3	1.43 (1.00–2.01)	86	1422	**0.046**	0.09	│───────●────────
NDVI 500 m	1	0.90 (0.15–5.58)	136	2267	0.91	0.95	──────────●│──────────
	2	0.96 (0.16–6.17)	134	2243	0.97	0.97	───────────●──────────
	3	1.83 (0.19–19.24)	90	1469	0.61	0.64	──────────│──●──────────────
NDVI 1500 m	1	0.94 (0.14–6.69)	136	2267	0.95	0.95	───────────●│───────────
	2	1.09 (0.15–8.25)	134	2243	0.93	0.97	───────────●────────────
	3	1.81 (0.16–22.96)	90	1469	0.64	0.64	───────────│──●───────────────

*Note*: OR for the association between residential LAI, NDVI, and risk of CeD within 500 and 1500 m buffers around the child's home, at age 3 years follow‐up in the CiPiS cohort. Model 1 = crude estimates. Model 2 = adjusted for sex, maternal age at delivery, season of birth, and maternal smoking during pregnancy. Model 3 = fully adjusted for all available covariates (see Table S1). Bold values indicate statistical significance at *p* < 0.05 and FDR adjustement *(q* < 0.05).

Abbreviations: CeD, celiac disease; CI, confidence interval; CiPiS, Celiac Prediction in Skåne; LAI, Leaf Area Index; NDVI, Normalized Difference Vegetation Index; OR, odds ratio.

At age 9, the association between LAI and CeD risk remained statistically significant in all three models at the 500 m radius. In the 1500 m radius, all the models also reached statistical significance, except in the crude model after correction for multiple testing (Table 2).

**Table 2:** Association of residential greenness at age 9 years and CeD in follow‐up.

Exposure	Model	OR (95% CI)	Cases	Controls	*p*‐Value	*p*‐ Adj	Forest
LAI 500 m	1	1.58 (1.11–2.21)	74	2033	**0.01**	**0.03**	│ ────────●───────
	2	1.62 (1.13–2.27)	73	2003	**0.01**	**0.03**	│ ────────●────────
	3	1.90 (1.23–2.87)	43	1315	**0.003**	**0.01**	│ ──────────●─────────
LAI 1500 m	1	1.48 (1.03–2.10)	81	2252	**0.03**	0.06	│────────●────────
	2	1.54 (1.07–2.20)	79	2217	**0.02**	**0.04**	│ ────────●────────
	3	1.90 (1.20–2.96)	45	1450	**0.005**	**0.01**	│ ──────────●──────────
NDVI 500 m	1	5.30 (0.44–65.78)	82	2292	0.19	0.26	────│────────●──────────────
	2	6.52 (0.50–87.43)	80	2254	0.16	0.21	────│─────────●──────────────
	3	28.85 (0.97–886.99)	45	1468	0.05	0.07	│─────────────────●───────
NDVI 1500 m	1	4.14 (0.29–61.33)	82	2292	0.30	0.30	───────│───────●──────────────
	2	5.11 (0.32–82.97)	80	2254	0.25	0.25	──────│────────●───────────────
	3	18.01 (0.44–738.62)	45	1468	0.13	0.13	────│───────────────●─────────

*Note*: OR for the association between residential LAI, NDVI, and risk of CeD within 500 and 1500 m buffers around the child's home, at age 9 years follow‐up in the CiPiS cohort. Model 1 = crude estimates. Model 2 = adjusted for sex, maternal age at delivery, season of birth, and maternal smoking during pregnancy. Model 3 = fully adjusted for all available covariates (see Table S1). Bold values indicate statistical significance at *p* < 0.05 and FDR adjustement (*q* < 0.05).

Abbreviations: CeD, celiac disease; CI, confidence interval; CiPiS, Celiac Prediction in Skåne; LAI, Leaf Area Index; NDVI, Normalized Difference Vegetation Index; OR, odds ratio

NDVI showed positive associations with CeD risk at age 9 years, but none of these associations were statistically significant (Table 2).

At birth and at age 15 years, associations between LAI and CeD risk were also positive. However, statistical significance was observed only before FDR correction and was limited to the 1500 m radius in Model 1 at birth and 500 m radius in Model 1 and 2 at age 15 years. For NDVI, no clear association with CeD risk was observed, with estimates varying in direction across models and none reaching statistical significance. At the 15‐year follow‐up, the number of CeD cases was limited, and results at this time point should therefore be interpreted with caution (Tables S12, –S13).

Some covariates showed statistically significant associations in fully adjusted models; however, these findings were based on small subgroup sizes and were therefore interpreted cautiously and explored further in sensitivity and modification analysis (see Section 3.4 and Tables S10 and S14).

### 3.2 LAI and correlation to land cover classes in the 1500‐m radius

**Figure 2:**
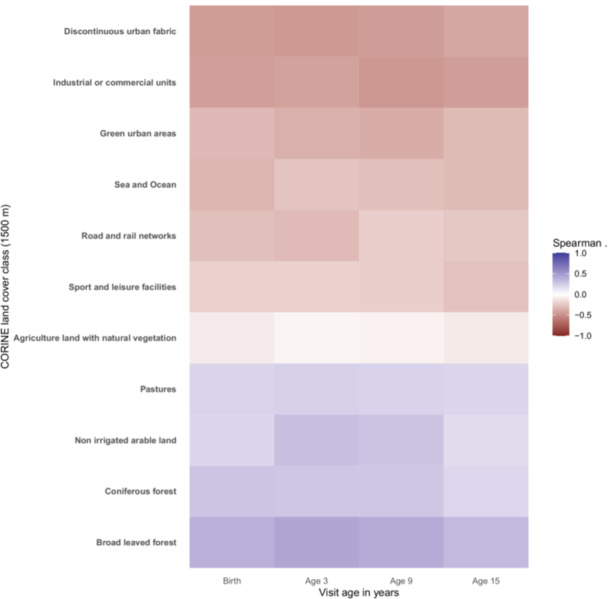
Spearman correlation between residential LAI and centered log‐ratio transformed CORINE land cover composition at 1500 m, shown descriptively by follow‐up age. Rare land cover classes were excluded (present in <5% of participants). CORINE, Coordination of Information on the Environment; LAI, Leaf Area Index.

To provide contextual interpretation of LAI, we calculated Spearman correlations between LAI at a 1500 m radius and CLR‐transformed CORINE Land Cover proportions. These correlations were used descriptively to illustrate which types of land cover were associated with higher or lower LAI values across follow‐up ages (Figure 2). Overall, higher LAI values corresponded, as expected, to forested and agricultural land cover, whereas lower LAI values were associated with artificial surfaces. Correlation patterns were broadly similar across time points.

### 3.3 No land cover category was clearly associated with the risk of CeD

The summaries of CORINE Land Cover categories among cases and controls at birth, ages 3, 9, and 15 years are available in Tables S2–S5. To estimate the association of residential land cover categories and CeD risk, we constructed logistic regression models in the same manner as mentioned above (Tables S6–S9). They showed that proximity to road and rail networks at the age of 3 and at the 1500 m radius was positively associated with CeD risk in all the models, but this was only statistically significant before FDR correction (Table S7).

At age 15 years, no CORINE land cover categories were robustly associated with CeD risk after correction for multiple testing (Table S9). Although point estimates in some categories suggested slightly increased odds ratios, the number of cases were very small (*n* = 10). The results are not interpreted further.

### 3.4 Sensitivity and effect modification analyses

Because some of the covariates appeared to influence the models, we conducted an effect modification analysis of the logistic regression, with LAI at a 1500 m radius as the main modifier. A possible interaction with maternal working status during pregnancy was observed (interaction *p* = 0.049, *p*. adj = 0.54, Table S10). However, the number of exposed cases was small (*n* = 7), and the finding should be interpreted with caution. In sensitivity analyses, removal of this variable from the adjusted model did not materially alter the estimated associations (Table S14). Overall, effect estimates remained stable across all model variants. The association between LAI and CeD was robust with only minor changes in odds ratios and *p*‐values. Excluding paternal education slightly strengthened the association, whereas removal of other variables had negligible impact.

## 4 DISCUSSION

In contrast to our initial hypothesis, the present study found that higher residential greenness, primarily assessed by LAI, was associated with an increased risk of CeD at ages 3 and 9 years. NDVI showed inconsistent and nonsignificant associations. In line with our hypothesis, proximity to road and rail networks was associated with a modestly elevated risk of CeD at age 3 years, although this association did not remain significant after FDR adjustment.

Our findings differ from several previous studies of immune‐mediated diseases. For example, Finnish children living in agricultural environments during early life have been reported to have a reduced risk of type 1 diabetes, potentially mediated through differences in microbial exposure.^6^ Similarly, lower access to green space has been associated with increased risk of atopy^7^ and inflammatory bowel disease.^9^ In contrast, urban living environments during infancy have been linked to higher risks of asthma and allergic sensitization, alongside differences in airway and gut microbiota.^5^ Together, these studies support the biodiversity hypothesis,^18^ which posits that exposure to more diverse natural environments may protect against immune‐mediated diseases.

However, our findings among children in Skåne do not support a protective effect of residential greenness for CeD. This discrepancy may reflect that not all green environments confer similar biological exposures. Indeed, previous studies have reported increased risk of wheezing, asthma, and allergic rhinitis in children living near coniferous‐dominated green spaces,^8^ illustrating that vegetation type and land use context may be critical in shaping health effects.

CeD incidence has previously been shown to vary geographically within Sweden.^19^ Skåne is characterized by extensive agricultural land use, with cereal crop cultivation representing a major proportion of land cover.^20^ In the present study, high LAI values primarily corresponded to agricultural areas and forests, indicating that the MODIS‐derived LAI metric captured biologically meaningful vegetation patterns at the landscape scale. Thus, LAI in this setting likely reflects a mixture of dense agricultural vegetation and forest land.

Analyses of CORINE land cover categories revealed no consistent associations between specific land cover types and CeD risk after correction for multiple testing, and there were no clear associations with arable land. These findings suggest that proximity to crop fields alone is unlikely to explain the observed associations with LAI. Future studies incorporating crop‐specific information and higher‐resolution land cover data may help clarify whether particular agricultural practices or vegetation types are relevant for CeD risk.

Although the present findings are inconsistent with the biodiversity hypothesis, they do not exclude the possibility that nonvegetational environmental factors may contribute to the observed associations. For example, viral exposures have been proposed as potential triggers of CeD,^21^ and environmental characteristics associated with agricultural or forested areas, such as patterns of animal contact or land use, could influence exposure pathways not captured by greenness metrics alone. However, such mechanisms remain speculative and require further investigation.

The limited number of cases, especially at later follow‐up ages, reduced the statistical precision for modest associations. Borderline or nonsignificant findings, particularly in stratified and sensitivity analyses, should therefore be interpreted with caution.

Some residential and socioeconomic factors may act as confounders. An inverse association between maternal employment outside the home during pregnancy and CeD risk was observed in certain models, but this finding was based on small numbers and did not show consistent effect modification. Sensitivity analyses indicated that exclusion of this variable did not materially alter the main associations, suggesting that this result likely reflects residual variation rather than a causal relationship.

LAI and NDVI are well‐established satellite‐based measures of vegetation derived from validated MODIS products. However, the spatial resolution of these products (250–500 m) implies that individual pixels often represent mixed land cover, limiting the ability to distinguish dense vegetation in small areas from more homogeneous vegetation across buffer zones. In addition, the MODIS LAI product is primarily optimized for forested environments, which may reduce precision in predominantly agricultural landscapes such as Skåne.

Nevertheless, the combined use of greenness indices and CORINE land cover data provides complementary information on vegetation density and land use, extending beyond NDVI‐only approaches commonly applied in studies of immune‐mediated diseases.^22^ Future studies using higher‐resolution satellite data and more advanced spatial methods may further refine exposure assessment.

Finally, the present analyses are based on CeD cases identified through repeated population‐based screening within the CiPiS cohort. Linkage to the Swedish National CeD Register indicates that most children diagnosed with CeD in Skåne during the study period were identified through the CiPiS screening.^12^ This population‐based design reduces bias related to healthcare‐seeking behavior. Nevertheless, as participation in screening was voluntary, the findings may not be fully generalizable to cases detected through routine clinical care.

Residual confounding and residential mobility between follow‐up time points may have resulted in exposure misclassification. Residential exposure was based on the parental address recorded in the population register, with no additional information available on time spent at alternative residences. However, the age‐specific cross‐sectional design partly addressed this limitation by basing each exposure assessment on the address recorded at the corresponding time point. Future studies incorporating longitudinal residential histories and additional environmental covariates, such as pet ownership, daycare attendance, and yard environment,^23^, ^24^, ^25^, ^26^ as well as satellite maps at higher spatio‐temporal resolutions^27^ would be valuable.

## 5 CONCLUSIONS

In this exploratory observational study, higher environmental greenness, as measured by LAI, was associated with an increased risk of CeD in childhood. Replication in independent cohorts is needed to assess the robustness and generalizability of this finding. If corroborated, future studies may then address potential environmental factors linked to greenness involved in the etiology of CeD.

## CONFLICT OF INTEREST STATEMENT

The authors declare no conflicts of interest.

## Supporting information

Supplemental Table S1 (3).

Supplemental Table S2 (2).

Supplemental Table S3 (2).

Supplemental Table S4 (2).

Supplemental Table S5 (1).

Supplemental Table S6 (1).

Supplemental Table S7 (1).

Supplemental Table S8 (1).

Supplemental Table S9 (1).

Supplemental Table S10 (1).

Supplemental Table S11 (1).

Supplemental Table S12 (1).

Supplemental Table S13 (1).

Supplemental Table S14 (1).

## Data Availability

All data needed to support the conclusions of this manuscript are included in the main text, tables, and figures. Restrictions defined in the General Data Protection Regulation (EU 2016/679) and the Finnish Data Protection Act 1050/2018 apply to the availability of sensitive data. Therefore, sensitive data is not publicly available.
