# Supplementary material for: Associations between residential greenness, land cover and risk of celiac disease in genetically at‐risk children: Celiac Prediction in Skåne study
Source: J Pediatr Gastroenterol Nutr. 2026 Apr 22;83(1):127–34. doi: 10.1002/jpn3.70440 (PMC13342773; doi:10.1002/jpn3.70440)
Supplement: Supplementary file 1 — Supplemental Table S1 (3). [file JPN3-83-127-s005.docx]

| ***Supplemental Table S1.* Covariates in the CiPiS study among children at HLA risk.** | | | | |
| --- | --- | --- | --- | --- |
| **All=2535** |  | **Cases**  **N=138 (100%)** | **Controls**  **N= 2397(100%)** | **p** |
| **Female (n (%))** |  | 85 (61.6) | 1145 (47.8) | **0.002^** |
| **Season of birth (n (%))** | *Spring* | 32 (23.2) | 510 (21.2) | 0.11^ |
|  | *Summer* | 53 (38.4) | 793 (33.1) |  |
|  | *Fall* | 36 (26.1) | 597 (24.9) |  |
|  | *Winter* | 17 (12.3) | 497 (20.7) |  |
| **Mother´s age at delivery**  **(Mean (SD))** |  | 31.9 (4.6) | 31.3(4.6) | 0.15¨ |
| **Mother smoking during pregnancy (n (%))** | *Yes* | 8 (5.8) | 196 (8.2) | 0.41^ |
|  | *No* | 127 (92.0) | 2161 (90.2) |  |
|  | *Missing* | 3 (2.2) | 40 (1.7) |  |
| **Mother smoking after pregnancy**  **(n (%))** | *Yes* | 11 (8.0) | 156 (6.5) | 0.62^ |
|  | *No* | 86 (62.3) | 1523 (63.5) |  |
|  | *Missing* | 41 (29.7) | 718 (30.0) |  |
| **Maternal educational level (n (%))** | *Elementary/High school* | 70 (50.7) | 1217 (50.7) | 1^ |
|  | *University* | 66 (47.8) | 1139 (47.5) |  |
|  | *Missing* | 2 (1.4) | 41 (1.7) |  |
| **Mother working away from home during pregnancy (n (%))** | *Yes* | 112 (81.2) | 2155 (89.9) | **0.001^** |
|  | *No* | 23 (16.7) | 201 (8.4) |  |
|  | *Missing* | 3 (2.2) | 41 (1.7) |  |
| **Mother born in Sweden (n (%))** | *Yes* | 123 (89.1) | 2163 (90.2) | 0.40^ |
|  | *No* | 13 (9.4) | 169 (8.2) |  |
|  | *Missing* | 2 (1.4) | 65 (2.7) |  |
| **Father smoking* (n (%))** | *Yes* | 9 (6.5) | 149 (6.2) | 1^ |
|  | *No* | 84(60.9) | 1441 (60.1) |  |
|  | *Missing* | 45 (32.6) | 807 (33.7) |  |
| **Paternal educational level* (n (%))** | *Elementary/High school* | 89 (64.5) | 1462 (61.0) | 0.46^ |
|  | *University* | 46 (33.3) | 883 (36.8) |  |
|  | *Missing* | 3 (2.2) | 52 (2.2) |  |
| **Father born in Sweden* (n (%))** | *Yes* | 128(92.8) | 2144 (89.4) | 0.29^ |
|  | *No* | 8(5.8) | 209(8.7) |  |
|  | *Missing* | 2 (1.4) | 44 (1.8) |  |

The available covariates in the study of CiPiS**.** *Reported by the father; completed using the mother´s response if missing, ¨t test. ^ Chi-square test. Cases consisted of children ever diagnosed with celiac disease in CiPiS, and controls of those who were not.
