# Supplementary material for: Associations between residential greenness, land cover and risk of celiac disease in genetically at‐risk children: Celiac Prediction in Skåne study
Source: J Pediatr Gastroenterol Nutr. 2026 Apr 22;83(1):127–34. doi: 10.1002/jpn3.70440 (PMC13342773; doi:10.1002/jpn3.70440)
Supplement: Supplementary file 2 — Supplemental Table S2 (2). [file JPN3-83-127-s004.docx]

| ***Supplemental Table S2*. Summary of CORINE Land Cover Categories at birth in the CiPiS study, comparing controls and cases of celiac disease.** | | | | | | |
| --- | --- | --- | --- | --- | --- | --- |
| **Birth** | **Control n=1666** | **Case n=108** |  |  |  |  |
| **Variable** | **Mean (SD)** | **Mean (SD)** | **Diff** | **SMD** | **p** | **p.adj** |
| Agriculture land with natural vegetation (1500 m) | 0.51 (2.91) | 0.17 (1.35) | -0.35 | -0.12 | **0.02** | 0.63 |
| Broad leaved forest (500 m) | 1.57 (7.64) | 2.27 (9.80) | 0.69 | 0.09 | 0.47 | 0.88 |
| Broad leaved forest (1500 m) | 3.11 (8.81) | 4.44 (10.74) | 1.33 | 0.15 | 0.21 | 0.77 |
| Coniferous forest (500 m) | 1.85 (9.89) | 2.39 (12.45) | 0.54 | 0.05 | 0.66 | 0.92 |
| Coniferous forest (1500 m) | 3.16 (10.51) | 3.66 (12.23) | 0.5 | 0.05 | 0.68 | 0.92 |
| Continuous urban fabric (500 m) | 0.67 (5.58) | 0.95 (5.73) | 0.29 | 0.05 | 0.62 | 0.92 |
| Continuous urban fabric (1500 m) | 0.40 (2.51) | 0.57 (2.38) | 0.17 | 0.07 | 0.47 | 0.88 |
| Discontinuous urban fabric (500 m) | 67.59 (33.46) | 61.81 (36.41) | -5.78 | -0.17 | 0.11 | 0.69 |
| Discontinuous urban fabric (1500 m) | 46.75 (26.28) | 41.38 (26.90) | -5.37 | -0.20 | 0.05 | 0.69 |
| Green urban areas (500 m) | 2.52 (9.40) | 2.94 (9.44) | 0.42 | 0.05 | 0.65 | 0.92 |
| Green urban areas (1500 m) | 3.61 (7.54) | 3.44 (7.58) | -0.17 | -0.02 | 0.82 | 0.92 |
| Industrial or commercial units (500 m) | 2.04 (7.85) | 1.38 (5.46) | -0.66 | -0.09 | 0.24 | 0.77 |
| Industrial or commercial units (1500 m) | 3.94 (8.45) | 3.99 (7.96) | 0.05 | 0.01 | 0.95 | 0.96 |
| Mineral extraction sites (1500 m) | 0.03 (0.52) | 0.00 (0.00) | -0.03 | -0.06 | **0.02** | 0.63 |
| Non irrigated arable land (500 m) | 19.63 (30.98) | 23.08 (33.51) | 3.46 | 0.11 | 0.30 | 0.79 |
| Non irrigated arable land (1500 m) | 30.01 (29.63) | 32.82 (31.08) | 2.81 | 0.09 | 0.36 | 0.81 |
| Pastures (500 m) | 1.46 (6.97) | 2.58 (9.19) | 1.12 | 0.16 | 0.21 | 0.77 |
| Pastures (1500 m) | 2.70 (6.34) | 3.56 (7.91) | 0.87 | 0.13 | 0.27 | 0.77 |
| Port areas (1500 m) | 0.32 (3.03) | 0.32 (1.45) | 0.01 | 0.00 | 0.97 | 0.97 |
| Road and rail networks (500 m) | 0.65 (3.75) | 0.77 (3.76) | 0.12 | 0.03 | 0.75 | 0.92 |
| Road and rail networks (1500 m) | 1.04 (3.01) | 1.17 (3.05) | 0.12 | 0.04 | 0.68 | 0.92 |
| Sea and Ocean (500 m) | 0.41 (3.09) | 0.46 (2.81) | 0.05 | 0.02 | 0.85 | 0.92 |
| Sea and Ocean (1500 m) | 1.92 (7.02) | 1.86 (6.97) | -0.06 | -0.01 | 0.93 | 0.96 |
| Sport and leisure facilities (1500 m) | 0.94 (3.59) | 0.64 (2.70) | -0.30 | -0.09 | 0.27 | 0.77 |
| Water bodies (1500 m) | 0.37 (2.68) | 0.56 (2.89) | 0.19 | 0.07 | 0.52 | 0.88 |
| Level 1 — Agricultural areas (500 m) | 20.93 (31.63) | 26.47 (35.79) | 5.54 | 0.17 | 0.12 | 0.69 |
| Level 1 — Agricultural areas (1500 m) | 33.10 (30.43) | 36.64 (32.29) | 3.54 | 0.12 | 0.27 | 0.77 |
| Level 1 — Artificial surfaces (500 m) | 74.89 (33.15) | 68.92 (37.28) | -5.97 | -0.18 | 0.11 | 0.69 |
| Level 1 — Artificial surfaces (1500 m) | 57.61 (31.28) | 52.20 (33.12) | -5.41 | -0.17 | 0.10 | 0.69 |
| Level 1 — Forest and semi natural areas (500 m) | 3.67 (12.98) | 4.15 (14.85) | 0.47 | 0.04 | 0.75 | 0.92 |
| Level 1 — Forest and semi natural areas (1500 m) | 6.82 (15.33) | 8.44 (16.69) | 1.62 | 0.11 | 0.33 | 0.81 |
| Level 1 — Water bodies (500 m) | 0.49 (3.28) | 0.46 (2.81) | -0.02 | -0.01 | 0.93 | 0.96 |
| Level 1 — Water bodies (1500 m) | 2.29 (7.42) | 2.49 (7.41) | 0.19 | 0.03 | 0.79 | 0.92 |
| Level 1 — Wetlands (1500 m) | 0.19 (1.67) | 0.24 (2.44) | 0.05 | 0.03 | 0.83 | 0.92 |
| Level 2 — Forests (500 m) | 4.99 (17.75) | 6.48 (21.83) | 1.49 | 0.08 | 0.49 | 0.88 |
| Level 2 — Forests (1500 m) | 11.13 (24.23) | 14.31 (28.17) | 3.19 | 0.13 | 0.25 | 0.77 |
| Level 2 — Urban fabric Industrial and construction sites (500 m) | 83.30 (32.72) | 76.43 (38.42) | -6.86 | -0.21 | 0.07 | 0.69 |
| Level 2 — Urban fabric Industrial and construction sites (1500 m) | 78.69 (30.00) | 71.97 (35.35) | -6.72 | -0.22 | 0.06 | 0.69 |
| Level 2 — Urban green spaces (500 m) | 3.01 (10.81) | 3.20 (10.28) | 0.19 | 0.02 | 0.86 | 0.92 |
| Level 2 — Urban green spaces (1500 m) | 5.86 (11.15) | 5.38 (11.16) | -0.48 | -0.04 | 0.67 | 0.92 |

Diff indicates the raw mean difference. SMD indicates the standardized mean difference. Reported p-values are from two-sided Welch´s t tests and were adjusted using the Benjamini-Hochberg false discovery rate procedure.
