# Supplementary material for: Associations between residential greenness, land cover and risk of celiac disease in genetically at‐risk children: Celiac Prediction in Skåne study
Source: J Pediatr Gastroenterol Nutr. 2026 Apr 22;83(1):127–34. doi: 10.1002/jpn3.70440 (PMC13342773; doi:10.1002/jpn3.70440)
Supplement: Supplementary file 3 — Supplemental Table S3 (2). [file JPN3-83-127-s010.docx]

| ***Supplemental Table S3.* Summary of CORINE Land Cover Categories at the 3-year follow-up in the CiPiS study, comparing controls and cases of celiac disease.** | | | | | | |
| --- | --- | --- | --- | --- | --- | --- |
| **Age 3 years** | **Control n=2267** | **Case n=136** |  |  |  |  |
| **Variable** | **Mean (SD)** | **Mean (SD)** | **Diff** | **SMD** | **p** | **p.adj** |
| Agriculture land with natural vegetation (1500 m) | 0.51 (2.86) | 0.51 (2.96) | 0.00 | 0.00 | 0.99 | 0.99 |
| Broad leaved forest (500 m) | 1.41 (7.09) | 2.59 (12.18) | 1.18 | 0.16 | 0.27 | 0.61 |
| Broad leaved forest (1500 m) | 2.81 (8.32) | 4.17 (11.31) | 1.36 | 0.16 | 0.17 | 0.61 |
| Coniferous forest (500 m) | 1.56 (9.03) | 1.38 (8.48) | -0.18 | -0.02 | 0.81 | 0.93 |
| Coniferous forest (1500 m) | 2.63 (9.31) | 3.44 (10.78) | 0.81 | 0.09 | 0.39 | 0.66 |
| Continuous urban fabric (500 m) | 0.81 (6.11) | 0.97 (7.08) | 0.16 | 0.03 | 0.8 | 0.93 |
| Continuous urban fabric (1500 m) | 0.65 (3.41) | 0.72 (3.62) | 0.07 | 0.02 | 0.83 | 0.93 |
| Discontinuous urban fabric (500 m) | 69.21 (32.36) | 65.68 (35.54) | -3.53 | -0.11 | 0.26 | 0.61 |
| Discontinuous urban fabric (1500 m) | 47.33 (25.93) | 43.59 (27.36) | -3.74 | -0.14 | 0.12 | 0.61 |
| Green urban areas (500 m) | 2.34 (8.99) | 1.66 (7.96) | -0.69 | -0.08 | 0.33 | 0.63 |
| Green urban areas (1500 m) | 3.57 (7.32) | 3.74 (7.64) | 0.17 | 0.02 | 0.80 | 0.93 |
| Mineral extraction sites (1500 m) | 0.09 (0.97) | 0.02 (0.23) | -0.06 | -0.07 | **0.03** | 0.61 |
| Industrial or commercial units (500 m) | 1.76 (7.64) | 1.63 (6.06) | -0.12 | -0.02 | 0.82 | 0.93 |
| Industrial or commercial units (1500 m) | 3.79 (8.18) | 3.67 (6.94) | -0.11 | -0.01 | 0.86 | 0.93 |
| Non irrigated arable land (500 m) | 18.66 (29.52) | 21.14 (32.17) | 2.48 | 0.08 | 0.38 | 0.66 |
| Non irrigated arable land (1500 m) | 29.73 (29.13) | 30.78 (30.47) | 1.05 | 0.04 | 0.69 | 0.89 |
| Pastures (500 m) | 1.69 (7.26) | 2.60 (10.16) | 0.91 | 0.12 | 0.31 | 0.62 |
| Pastures (1500 m) | 2.76 (6.56) | 3.56 (7.43) | 0.8 | 0.12 | 0.22 | 0.61 |
| Port areas (1500 m) | 0.30 (2.08) | 0.31 (2.25) | 0.02 | 0.01 | 0.94 | 0.97 |
| Road and rail networks (500 m) | 0.67 (3.68) | 1.17 (4.62) | 0.50 | 0.13 | 0.22 | 0.61 |
| Road and rail networks (1500 m) | 1.33 (3.51) | 2.24 (4.38) | 0.91 | 0.25 | **0.02** | 0.54 |
| Sea and Ocean (500 m) | 0.34 (2.76) | 0.24 (2.02) | -0.11 | -0.04 | 0.56 | 0.81 |
| Sea and Ocean (1500 m) | 1.85 (6.79) | 1.07 (5.91) | -0.78 | -0.12 | 0.14 | 0.61 |
| Sport and leisure facilities (1500 m) | 1.15 (4.11) | 0.76 (2.81) | -0.39 | -0.10 | 0.13 | 0.61 |
| Water bodies (1500 m) | 0.40 (3.13) | 0.35 (2.33) | -0.05 | -0.02 | 0.80 | 0.93 |
| Level 1 — Agricultural areas (500 m) | 20.83 (30.98) | 24.69 (34.26) | 3.86 | 0.12 | 0.2 | 0.61 |
| Level 1 — Agricultural areas (1500 m) | 33.19 (30.32) | 35.24 (31.57) | 2.05 | 0.07 | 0.46 | 0.71 |
| Level 1 — Artificial surfaces (500 m) | 75.36 (32.56) | 71.11 (36.79) | -4.26 | -0.13 | 0.19 | 0.61 |
| Level 1 — Artificial surfaces (1500 m) | 58.40 (31.52) | 55.21 (33.99) | -3.19 | -0.10 | 0.29 | 0.62 |
| Level 1 — Forest and semi natural areas (500 m) | 3.33 (12.31) | 3.97 (15.55) | 0.64 | 0.05 | 0.64 | 0.88 |
| Level 1 — Forest and semi natural areas (1500 m) | 6.03 (14.42) | 7.91 (17.65) | 1.88 | 0.13 | 0.23 | 0.61 |
| Level 1 — Water bodies (500 m) | 0.44 (3.09) | 0.24 (2.02) | -0.21 | -0.07 | 0.26 | 0.61 |
| Level 1 — Water bodies (1500 m) | 2.26 (7.38) | 1.44 (6.30) | -0.82 | -0.11 | 0.15 | 0.61 |
| Level 1 — Wetlands (1500 m) | 0.13 (1.43) | 0.20 (2.18) | 0.07 | 0.05 | 0.70 | 0.89 |
| Level 2 — Forests (500 m) | 4.62 (17.23) | 6.28 (22.41) | 1.66 | 0.09 | 0.4 | 0.66 |
| Level 2 — Forests (1500 m) | 9.90 (23.07) | 12.90 (27.51) | 3.00 | 0.13 | 0.21 | 0.61 |
| Level 2 — Urban fabric Industrial and construction sites (500 m) | 83.89 (32.17) | 79.35 (38.30) | -4.54 | -0.14 | 0.18 | 0.61 |
| Level 2 — Urban fabric Industrial and construction sites (1500 m) | 79.49 (29.47) | 73.97 (34.45) | -5.52 | -0.19 | 0.07 | 0.61 |
| Level 2 — Urban green spaces (500 m) | 3.24 (11.45) | 1.87 (9.55) | -1.37 | -0.12 | 0.11 | 0.61 |
| Level 2 — Urban green spaces (1500 m) | 6.42 (12.60) | 5.78 (10.84) | -0.64 | -0.05 | 0.51 | 0.76 |

Diff indicates the raw mean difference. SMD indicates the standardized mean difference. Reported p-values are from two-sided Welch´s t tests and were adjusted using the Benjamini-Hochberg false discovery rate procedure.
