# Supplementary material for: Associations between residential greenness, land cover and risk of celiac disease in genetically at‐risk children: Celiac Prediction in Skåne study
Source: J Pediatr Gastroenterol Nutr. 2026 Apr 22;83(1):127–34. doi: 10.1002/jpn3.70440 (PMC13342773; doi:10.1002/jpn3.70440)
Supplement: Supplementary file 4 — Supplemental Table S4 (2). [file JPN3-83-127-s002.docx]

| ***Supplemental Table S4.* Summary of CORINE Land Cover Categories at the 9-year follow-up in the CiPiS study, comparing controls and cases of celiac disease.** | | | | | | |
| --- | --- | --- | --- | --- | --- | --- |
| **Age 9 years** | **Control n=2295** | **Case n=82** |  |  |  |  |
| **Variable** | **Mean (SD)** | **Mean (SD)** | **Diff** | **SMD** | **p** | **p.adj** |
| Agriculture land with natural vegetation (1500 m) | 0.57 (2.94) | 0.52 (3.10) | -0.06 | -0.02 | 0.87 | 0.96 |
| Broad leaved forest (500 m) | 1.46 (6.92) | 2.99 (12.37) | 1.52 | 0.21 | 0.27 | 0.95 |
| Broad leaved forest (1500 m) | 2.77 (8.19) | 4.65 (11.88) | 1.88 | 0.23 | 0.16 | 0.83 |
| Coniferous forest (500 m) | 1.41 (8.15) | 1.20 (6.30) | -0.21 | -0.03 | 0.77 | 0.95 |
| Coniferous forest (1500 m) | 2.53 (8.87) | 3.71 (12.68) | 1.18 | 0.13 | 0.41 | 0.95 |
| Continuous urban fabric (500 m) | 0.45 (5.14) | 0.81 (7.36) | 0.37 | 0.07 | 0.66 | 0.95 |
| Continuous urban fabric (1500 m) | 0.40 (2.96) | 0.41 (2.24) | 0.01 | 0.00 | 0.97 | 0.98 |
| Discontinuous urban fabric (500 m) | 70.49 (31.73) | 68.93 (32.36) | -1.56 | -0.05 | 0.67 | 0.95 |
| Discontinuous urban fabric (1500 m) | 47.72 (25.63) | 45.79 (24.90) | -1.93 | -0.08 | 0.49 | 0.95 |
| Green urban areas (500 m) | 2.01 (8.32) | 1.59 (7.22) | -0.42 | -0.05 | 0.61 | 0.95 |
| Green urban areas (1500 m) | 3.06 (6.73) | 3.91 (7.45) | 0.85 | 0.13 | 0.31 | 0.95 |
| Industrial or commercial units (500 m) | 1.76 (7.80) | 1.57 (5.91) | -0.19 | -0.02 | 0.78 | 0.95 |
| Industrial or commercial units (1500 m) | 4.06 (8.23) | 3.16 (6.89) | -0.90 | -0.11 | 0.25 | 0.95 |
| Mineral extraction sites (1500 m) | 0.09 (1.06) | 0.01 (0.05) | -0.09 | -0.08 | **0.0001** | **0.002** |
| Non irrigated arable land (500 m) | 18.56 (28.58) | 17.86 (28.81) | -0.70 | -0.02 | 0.83 | 0.95 |
| Non irrigated arable land (1500 m) | 30.55 (28.49) | 30.55 (29.40) | 0.00 | 0.00 | 1.00 | 1.00 |
| Pastures (500 m) | 1.66 (7.32) | 2.12 (8.75) | 0.47 | 0.06 | 0.63 | 0.95 |
| Pastures (1500 m) | 2.87 (6.76) | 3.22 (7.72) | 0.34 | 0.05 | 0.69 | 0.95 |
| Port areas (1500 m) | 0.29 (2.79) | 0.04 (0.31) | -0.25 | -0.09 | **0.0003** | **0.004** |
| Road and rail networks (500 m) | 0.13 (1.68) | 0.25 (2.30) | 0.13 | 0.08 | 0.62 | 0.95 |
| Road and rail networks (1500 m) | 0.40 (2.25) | 0.45 (1.88) | 0.05 | 0.02 | 0.81 | 0.95 |
| Sea and Ocean (500 m) | 0.34 (2.65) | 0.00 (0.00) | -0.34 | -0.13 | **0.0001** | **0.0001** |
| Sea and Ocean (1500 m) | 2.02 (7.03) | 0.85 (4.16) | -1.17 | -0.17 | 0.02 | 0.17 |
| Sport and leisure facilities (1500 m) | 1.38 (4.54) | 1.50 (4.66) | 0.12 | 0.03 | 0.82 | 0.95 |
| Water bodies (1500 m) | 0.35 (3.01) | 0.26 (1.83) | -0.09 | -0.03 | 0.67 | 0.95 |
| Level 1 — Agricultural areas (500 m) | 20.82 (30.09) | 21.28 (30.43) | 0.47 | 0.02 | 0.89 | 0.96 |
| Level 1 — Agricultural areas (1500 m) | 34.18 (29.53) | 34.62 (29.55) | 0.44 | 0.02 | 0.89 | 0.96 |
| Level 1 — Artificial surfaces (500 m) | 75.50 (31.68) | 73.61 (32.78) | -1.89 | -0.06 | 0.61 | 0.95 |
| Level 1 — Artificial surfaces (1500 m) | 57.50 (30.36) | 55.23 (29.75) | -2.27 | -0.07 | 0.50 | 0.95 |
| Level 1 — Forest and semi natural areas (500 m) | 3.20 (11.85) | 4.96 (17.03) | 1.76 | 0.15 | 0.36 | 0.95 |
| Level 1 — Forest and semi natural areas (1500 m) | 5.84 (13.88) | 8.66 (18.55) | 2.82 | 0.2 | 0.18 | 0.83 |
| Level 1 — Water bodies (500 m) | 0.46 (3.07) | 0.15 (1.38) | -0.31 | -0.1 | 0.06 | 0.4 |
| Level 1 — Water bodies (1500 m) | 2.38 (7.56) | 1.21 (4.52) | -1.17 | -0.16 | **0.03** | 0.21 |
| Level 1 — Wetlands (1500 m) | 0.11 (1.33) | 0.29 (2.59) | 0.17 | 0.12 | 0.55 | 0.95 |
| Level 2 — Forests (500 m) | 4.51 (16.91) | 6.82 (21.42) | 2.31 | 0.14 | 0.34 | 0.95 |
| Level 2 — Forests (1500 m) | 9.72 (22.57) | 11.26 (23.11) | 1.54 | 0.07 | 0.55 | 0.95 |
| Level 2 — Urban fabric Industrial and construction sites (500 m) | 84.94 (31.13) | 83.53 (32.39) | -1.42 | -0.05 | 0.70 | 0.95 |
| Level 2 — Urban fabric Industrial and construction sites (1500 m) | 79.83 (29.14) | 74.57 (31.67) | -5.26 | -0.18 | 0.14 | 0.83 |
| Level 2 — Urban green spaces (500 m) | 2.97 (10.79) | 2.34 (7.94) | -0.63 | -0.06 | 0.49 | 0.95 |
| Level 2 — Urban green spaces (1500 m) | 6.31 (12.66) | 6.85 (10.53) | 0.54 | 0.04 | 0.65 | 0.95 |

Diff indicates the raw mean difference. SMD indicates the standardized mean difference. Reported p-values are from two-sided Welch´s t tests and were adjusted using the Benjamini-Hochberg false discovery rate procedure.
