# Supplementary material for: Associations between residential greenness, land cover and risk of celiac disease in genetically at‐risk children: Celiac Prediction in Skåne study
Source: J Pediatr Gastroenterol Nutr. 2026 Apr 22;83(1):127–34. doi: 10.1002/jpn3.70440 (PMC13342773; doi:10.1002/jpn3.70440)
Supplement: Supplementary file 5 — Supplemental Table S5 (1). [file JPN3-83-127-s003.docx]

| ***Supplemental Table S5****.* **Summary of CORINE Land Cover Categories at the 15-year follow-up in the CiPiS study, comparing controls and cases of celiac disease.** | | | | | | |
| --- | --- | --- | --- | --- | --- | --- |
| **Age 15 years** | **Control n=2278** | **Case n=10** |  |  |  |  |
| **Variable** | **Mean (SD)** | **Mean (SD)** | **Diff** | **SMD** | **p** | **p.adj** |
| Agriculture land with natural vegetation (1500 m) | 0.62 (3.06) | 0.00 (0.00) | -0.62 | -0.2 | **0.0001** | **0.0001** |
| Broad leaved forest (500 m) | 1.41 (6.91) | 10.00 (31.62) | 8.59 | 1.2 | 0.41 | 0.67 |
| Broad leaved forest (1500 m) | 2.78 (8.24) | 7.18 (22.71) | 4.40 | 0.53 | 0.56 | 0.67 |
| Coniferous forest (500 m) | 1.30 (8.09) | 4.17 (13.18) | 2.86 | 0.35 | 0.51 | 0.67 |
| Coniferous forest (1500 m) | 2.41 (8.87) | 5.49 (13.38) | 3.08 | 0.35 | 0.49 | 0.67 |
| Continuous urban fabric (500 m) | 0.63 (6.00) | 6.67 (21.08) | 6.04 | 0.99 | 0.39 | 0.67 |
| Continuous urban fabric (1500 m) | 0.51 (3.32) | 1.65 (5.22) | 1.14 | 0.34 | 0.51 | 0.67 |
| Discontinuous urban fabric (500 m) | 70.74 (31.61) | 59.26 (41.98) | -11.47 | -0.36 | 0.41 | 0.67 |
| Discontinuous urban fabric (1500 m) | 48.74 (25.76) | 45.13 (33.98) | -3.61 | -0.14 | 0.75 | 0.79 |
| Green urban areas (500 m) | 2.21 (8.64) | 0.00 (0.00) | -2.21 | -0.26 | **0.0001** | **0.0001** |
| Green urban areas (1500 m) | 3.16 (6.84) | 2.30 (3.95) | -0.85 | -0.12 | 0.52 | 0.67 |
| Industrial or commercial units (500 m) | 2.09 (8.70) | 2.05 (5.28) | -0.04 | 0.00 | 0.98 | 0.99 |
| Industrial or commercial units (1500 m) | 4.38 (8.59) | 7.08 (13.64) | 2.70 | 0.31 | 0.55 | 0.67 |
| Mineral extraction sites (1500 m) | 0.09 (1.33) | 0.00 (0.00) | -0.09 | -0.07 | **0.001** | **0.004** |
| Non irrigated arable land (500 m) | 17.56 (27.98) | 12.31 (31.65) | -5.25 | -0.19 | 0.61 | 0.73 |
| Non irrigated arable land (1500 m) | 28.96 (28.04) | 16.47 (28.18) | -12.49 | -0.45 | 0.20 | 0.66 |
| Pastures (500 m) | 1.72 (7.58) | 3.88 (10.88) | 2.15 | 0.28 | 0.55 | 0.67 |
| Pastures (1500 m) | 2.89 (6.93) | 5.74 (9.25) | 2.85 | 0.41 | 0.36 | 0.67 |
| Road and rail networks (500 m) | 0.13 (1.58) | 0.00 (0.00) | -0.12 | -0.08 | **0.002** | **0.0007** |
| Road and rail networks (1500 m) | 0.43 (2.34) | 0.00 (0.00) | -0.42 | -0.18 | **0.0001** | **0.0001** |
| Sea and Ocean (500 m) | 0.34 (2.77) | 1.67 (5.27) | 1.33 | 0.48 | 0.45 | 0.67 |
| Sea and Ocean (1500 m) | 1.94 (6.77) | 5.01 (13.65) | 3.07 | 0.45 | 0.49 | 0.67 |
| Sport and leisure facilities (1500 m) | 1.45 (4.78) | 2.22 (3.90) | 0.77 | 0.16 | 0.55 | 0.67 |
| Transitional woodland shrubs (1000 m) | 0.05 (0.82) | 0.00 (0.00) | -0.05 | -0.06 | **0.01** | **0.02** |
| Water bodies (1500 m) | 0.39 (3.04) | 0.00 (0.00) | -0.39 | -0.13 | **0.0001** | **0.0001** |
| Level 1 — Agricultural areas (500 m) | 19.86 (29.54) | 16.19 (34.52) | -3.68 | -0.12 | 0.74 | 0.79 |
| Level 1 — Agricultural areas (1500 m) | 32.64 (29.21) | 22.91 (30.79) | -9.72 | -0.33 | 0.34 | 0.67 |
| Level 1 — Artificial surfaces (500 m) | 76.66 (31.18) | 67.98 (41.16) | -8.68 | -0.28 | 0.52 | 0.67 |
| Level 1 — Artificial surfaces (1500 m) | 59.20 (30.38) | 59.27 (39.65) | 0.07 | 0.00 | 1.00 | 1.00 |
| Level 1 — Forest and semi natural areas (500 m) | 2.97 (11.38) | 14.17 (32.88) | 11.20 | 0.97 | 0.31 | 0.67 |
| Level 1 — Forest and semi natural areas (1500 m) | 5.69 (13.74) | 12.81 (28.75) | 7.12 | 0.51 | 0.45 | 0.67 |
| Level 1 — Water bodies (500 m) | 0.47 (3.25) | 1.67 (5.27) | 1.19 | 0.37 | 0.49 | 0.67 |
| Level 1 — Water bodies (1500 m) | 2.34 (7.32) | 5.01 (13.65) | 2.67 | 0.36 | 0.55 | 0.67 |
| Level 1 — Wetlands (1500 m) | 0.14 (1.40) | 0.00 (0.00) | -0.14 | -0.10 | **0.0001** | **0.0001** |
| Level 2 — Forests (500 m) | 4.18 (16.40) | 14.17 (32.88) | 9.99 | 0.61 | 0.36 | 0.67 |
| Level 2 — Forests (1500 m) | 9.54 (22.48) | 14.41 (33.11) | 4.87 | 0.22 | 0.65 | 0.75 |
| Level 2 — Urban fabric Industrial and construction sites (500 m) | 85.60 (30.39) | 75.83 (42.03) | -9.77 | -0.32 | 0.48 | 0.67 |
| Level 2 — Urban fabric Industrial and construction sites (1500 m) | 80.34 (28.70) | 70.12 (39.55) | -10.22 | -0.36 | 0.44 | 0.67 |
| Level 2 — Urban green spaces (500 m) | 3.24 (11.37) | 0.00 (0.00) | -3.24 | -0.29 | **0.0001** | **0.0001** |
| Level 2 — Urban green spaces (1500 m) | 6.39 (12.69) | 5.47 (6.57) | -0.92 | -0.07 | 0.67 | 0.76 |

Diff indicates the raw mean difference. SMD indicates the standardized mean difference. Reported p-values are from two-sided Welch´s t tests and were adjusted using the Benjamini-Hochberg false discovery rate procedure.
