# Supplementary material for: Associations between residential greenness, land cover and risk of celiac disease in genetically at‐risk children: Celiac Prediction in Skåne study
Source: J Pediatr Gastroenterol Nutr. 2026 Apr 22;83(1):127–34. doi: 10.1002/jpn3.70440 (PMC13342773; doi:10.1002/jpn3.70440)
Supplement: Supplementary file 6 — Supplemental Table S6 (1). [file JPN3-83-127-s008.docx]

| ***Supplemental Table S6.* Adjusted associations between prevalent CORINE land cover level 3 classes and celiac disease risk at birth among CiPiS participants.** | | | | | |
| --- | --- | --- | --- | --- | --- |
| **Label** | **OR (95% CI)** | **Cases** | **Controls** | **p.value** | **p.adj** |
| Broad leaved forest 500 m | 1.01 (0.99–1.03) | 106 | 1639 | 0.35 | 0.74 |
| Coniferous forest 500 m | 1.01 (0.99–1.02) | 106 | 1639 | 0.42 | 0.79 |
| Discontinuous urban fabric 500 m | 0.99 (0.99–1.00) | 106 | 1639 | 0.07 | 0.54 |
| Green urban areas 500 m | 1.00 (0.98–1.02) | 106 | 1639 | 0.61 | 0.87 |
| Industrial or commercial units 500 m | 0.99 (0.95–1.01) | 106 | 1639 | 0.37 | 0.74 |
| Non irrigated arable land 500 m | 1.00 (1.00–1.01) | 106 | 1639 | 0.29 | 0.74 |
| Pastures 500 m | 1.01 (0.99–1.03) | 106 | 1639 | 0.16 | 0.56 |
| Broad leaved forest 1500 m | 1.01 (0.99–1.03) | 106 | 1639 | 0.14 | 0.56 |
| Coniferous forest 1500 m | 1.01 (0.99–1.02) | 106 | 1639 | 0.48 | 0.79 |
| Discontinuous urban fabric 1500 m | 0.99 (0.98–1.00) | 106 | 1639 | 0.03 | 0.54 |
| Green urban areas 1500 m | 1.00 (0.97–1.02) | 106 | 1639 | 0.84 | 0.98 |
| Industrial or commercial units 1500 m | 1.00 (0.98–1.02) | 106 | 1639 | 0.85 | 0.98 |
| Non irrigated arable land 1500 m | 1.00 (1.00–1.01) | 106 | 1639 | 0.34 | 0.74 |
| Pastures 1500 m | 1.02 (0.99–1.04) | 106 | 1639 | 0.17 | 0.56 |
| Road and rail networks 1500 m | 1.00 (0.93–1.06) | 106 | 1639 | 0.96 | 0.98 |
| Sea and ocean 1500 m | 1.00 (0.97–1.02) | 106 | 1639 | 0.90 | 0.98 |

Logistic regression analyses were performed using the adjustment model (*Model 2*), including sex, season of birth, maternal age at delivery, and maternal smoking during pregnancy. CORINE land cover categories with mean coverage ≥1% were included in the table. Odds ratios (ORs) are reported per unit increase in proportional land cover within the specified buffer radius. P-values were adjusted for multiple testing using the Benjamini-Hochberg false discovery rate (FDR) procedure within each visit.
