# Supplementary material for: Associations between residential greenness, land cover and risk of celiac disease in genetically at‐risk children: Celiac Prediction in Skåne study
Source: J Pediatr Gastroenterol Nutr. 2026 Apr 22;83(1):127–34. doi: 10.1002/jpn3.70440 (PMC13342773; doi:10.1002/jpn3.70440)
Supplement: Supplementary file 10 — Supplemental Table S10 (1). [file JPN3-83-127-s009.docx]

| ***Supplemental Table S10.* Effect modification at age 9 years for celiac disease and leaf area index in the buffer 1500m radius** | | | | | |
| --- | --- | --- | --- | --- | --- |
| **Modifier** | **Stratum** | **Cases** | **OR (95% CI)** | **p interaction** | **p.adj** |
| Sex | Female | 27 | 2.02 (1.13–3.60) | 0.77 | 0.98 |
|  | Male | 18 | 1.75 (0.87–3.56) |  |  |
| Season of birth | Spring | 11 | 1.49 (0.57–3.90) | 0.28 | 0.68 |
|  | Summer | 18 | 1.64 (0.81–3.30) |  |  |
|  | Fall | 11 | 1.65 (0.62–4.36) |  |  |
|  | Winter | 5 | 5.80 (1.76–19.11) |  |  |
| Maternal smoking during pregnancy | No | 41 | 1.68 (1.04–2.73) | 0.12 | 0.67 |
|  | Yes | 4 | 5.65 (1.17–27.37) |  |  |
| Maternal smoking after pregnancy | No | 41 | 1.90 (1.19–3.04) | 0.98 | 0.98 |
|  | Yes | 4 | 1.94 (0.46–8.21) |  |  |
| Mother working away from home during pregnancy | No | 7 | 6.08 (1.56–23.78) | **0.049** | 0.54 |
|  | Yes | 38 | 1.57 (0.94–2.60) |  |  |
| Maternal educational level | Below university | 22 | 2.40 (1.30–4.43) | 0.29 | 0.68 |
|  | University | 23 | 1.47 (0.75–2.89) |  |  |
| Mother born in Sweden | No | 4 | 1.70 (0.37–7.86) | 0.88 | 0.98 |
|  | Yes | 41 | 1.93 (1.21–3.07) |  |  |
| Paternal smoking status | No | 43 | 1.85 (1.17–2.92) | 0.51 | 0.86 |
|  | Yes | 2 | 3.85 (0.45–33.03) |  |  |
| Paternal educational level | Below university | 32 | 2.07 (1.23–3.47) | 0.55 | 0.86 |
|  | University | 13 | 1.50 (0.59–3.79) |  |  |
| Father born in Sweden | No | 2 | 0.68 (0.07–6.39) | 0.31 | 0.68 |
|  | Yes | 43 | 2.02 (1.28–3.19) |  |  |
| Maternal age | <25 | 2 | 1.82 (0.27–12.09) | 0.93 | 0.98 |
|  | 25–34 | 33 | 1.82 (1.04–3.20) |  |  |
|  | ≥35 | 10 | 2.21 (0.97–5.05) |  |  |

Stratum-specific ORs are derived from a single interaction model in logistic regression with residential leaf area index in the 1500m radius as the main modifier among children in the CiPiS study; 'P interaction' is an omnibus likelihood-ratio test, p.adj is BH-adjusted p values across modifiers.
