# Supplementary material for: Associations between residential greenness, land cover and risk of celiac disease in genetically at‐risk children: Celiac Prediction in Skåne study
Source: J Pediatr Gastroenterol Nutr. 2026 Apr 22;83(1):127–34. doi: 10.1002/jpn3.70440 (PMC13342773; doi:10.1002/jpn3.70440)
Supplement: Supplementary file 11 — Supplemental Table S11 (1). [file JPN3-83-127-s007.docx]

| ***Supplemental Table S11.* Residential mean Leaf Area Index (LAI) and Normalized Difference Vegetation Index (NDVI) for the participants in the CiPiS study.** | | | | | | | |
| --- | --- | --- | --- | --- | --- | --- | --- |
|  | **Variable** | **Control** | **Case** | **Diff** | **SMD** | **p.value** | **p.adj** |
| **Birth** | LAI radius 500 m | 1.76 (0.64), n=1420 | 1.83 (0.69), n=100 | +0.08 | +0.12 | 0.29 | 0.59 |
|  | LAI radius 1500 m | 1.86 (0.62), n=1640 | 1.98 (0.73), n=105 | +0.12 | +0.19 | 0.10 | 0.41 |
|  | NDVI radius 500 m | 0.61 (0.09), n=1666 | 0.61 (0.10), n=108 | +0.00 | +0.02 | 0.83 | 0.83 |
|  | NDVI radius 1500 m | 0.62 (0.08), n=1666 | 0.63 (0.10), n=108 | +0.00 | +0.05 | 0.70 | 0.83 |
| **Age 3 years** | LAI radius 500 m | 1.71 (0.60), n=1928 | 1.87 (0.70), n=116 | +0.16 | +0.26 | **0.02** | 0.06 |
|  | LAI radius 1500 m | 1.83 (0.59), n=2191 | 1.96 (0.68), n=128 | +0.14 | +0.23 | **0.03** | 0.06 |
|  | NDVI radius 500 m | 0.60 (0.10), n=2267 | 0.60 (0.11), n=136 | -0.00 | -0.01 | 0.93 | 0.96 |
|  | NDVI radius 1500 m | 0.62 (0.09), n=2267 | 0.62 (0.11), n=136 | -0.00 | -0.01 | 0.96 | 0.96 |
| **Age 9 years** | LAI radius 500 m | 1.75 (0.59), n=2033 | 1.94 (0.72), n=74 | +0.19 | +0.31 | **0.03** | 0.12 |
|  | LAI radius 1500 m | 1.89 (0.58), n=2252 | 2.03 (0.67), n=81 | +0.14 | +0.25 | 0.06 | 0.12 |
|  | NDVI radius 500 m | 0.60 (0.09), n=2292 | 0.61 (0.09), n=82 | +0.01 | +0.15 | 0.21 | 0.28 |
|  | NDVI radius 1500 m | 0.61 (0.08), n=2292 | 0.62 (0.09), n=82 | +0.01 | +0.12 | 0.34 | 0.34 |
| **Age 15 years** | LAI radius 500 m | 1.58 (0.60), n=1961 | 2.06 (1.14), n=7 | +0.49 | +0.81 | 0.30 | 0.66 |
|  | LAI radius 1500 m | 1.68 (0.60), n=2201 | 1.93 (0.94), n=10 | +0.25 | +0.42 | 0.42 | 0.66 |
|  | NDVI radius 500 m | 0.59 (0.10), n=2243 | 0.61 (0.12), n=10 | +0.03 | +0.27 | 0.49 | 0.66 |
|  | NDVI radius 1500 m | 0.59 (0.09), n=2243 | 0.61 (0.09), n=10 | +0.01 | +0.14 | 0.67 | 0.67 |

Summary of greenness indices at birth and the follow-up at ages 3, 9 and 15 years with comparison between controls and cases of celiac disease in the CiPiS study. Diff indicates the raw mean difference. SMD indicates the standardized mean difference. Reported p-values are from two-sided Welch´s t tests and were adjusted using the Benjamini-Hochberg false discovery rate procedure.
