# Supplementary material for: Associations between residential greenness, land cover and risk of celiac disease in genetically at‐risk children: Celiac Prediction in Skåne study
Source: J Pediatr Gastroenterol Nutr. 2026 Apr 22;83(1):127–34. doi: 10.1002/jpn3.70440 (PMC13342773; doi:10.1002/jpn3.70440)
Supplement: Supplementary file 12 — Supplemental Table S12 (1). [file JPN3-83-127-s014.docx]

| ***Supplemental Table S12.* Association of residential greenness at birth and celiac disease in follow-up among CiPiS participants.** | | | | | | |
| --- | --- | --- | --- | --- | --- | --- |
| **Exposure** | **Model** | **OR (95% CI)** | **Cases** | **Controls** | **p.value** | **p.adj** |
| LAI 500 m | 1 | 1.19 (0.87–1.59) | 100 | 1420 | 0.26 | 0.52 |
|  | 2 | 1.21 (0.88–1.62) | 98 | 1395 | 0.22 | 0.45 |
|  | 3 | 1.09 (0.73–1.57) | 67 | 908 | 0.66 | 0.66 |
| LAI 1500 m | 1 | 1.33 (0.98–1.78) | 105 | 1640 | 0.06 | 0.23 |
|  | 2 | 1.36 (1.00–1.83) | 103 | 1614 | **0.04** | 0.18 |
|  | 3 | 1.17 (0.79–1.70) | 70 | 1055 | 0.42 | 0.56 |
| NDVI 500 m | 1 | 1.31 (0.16–11.53) | 108 | 1666 | 0.81 | 0.81 |
|  | 2 | 1.36 (0.15–12.56) | 106 | 1639 | 0.79 | 0.79 |
|  | 3 | 0.33 (0.02–4.92) | 73 | 1071 | 0.41 | 0.56 |
| NDVI 1500 m | 1 | 1.78 (0.16–20.52) | 108 | 1666 | 0.64 | 0.81 |
|  | 2 | 1.99 (0.17–24.16) | 106 | 1639 | 0.59 | 0.78 |
|  | 3 | 0.28 (0.01–5.96) | 73 | 1071 | 0.41 | 0.56 |

Odds ratio (OR) for the association between residential Leaf Area Index (LAI) and Normalized Difference Vegetation Index (NDVI) and risk of celiac disease within 500 m and 1500 m buffers around the child’s home, at birth in the CiPiS cohort. Model 1 = crude estimates. Model 2 = adjusted for sex, maternal age at delivery, season of birth, and maternal smoking during pregnancy. Model 3 = fully adjusted for all available covariates (see Supplemental Table S1).
