# Supplementary material for: Associations between residential greenness, land cover and risk of celiac disease in genetically at‐risk children: Celiac Prediction in Skåne study
Source: J Pediatr Gastroenterol Nutr. 2026 Apr 22;83(1):127–34. doi: 10.1002/jpn3.70440 (PMC13342773; doi:10.1002/jpn3.70440)
Supplement: Supplementary file 13 — Supplemental Table S13 (1). [file JPN3-83-127-s013.docx]

| ***Supplemental Table S13.* Association of residential greenness and celiac disease at age 15 years among CiPiS participants.** | | | | | | |
| --- | --- | --- | --- | --- | --- | --- |
| **Exposure** | **Model** | **OR (95% CI)** | **Cases** | **Controls** | **p.value** | **p.adj** |
| LAI 500 m | 1 | 2.56 (0.96–5.92) | 7 | 1961 | **0.04** | 0.15 |
|  | 2 | 2.41 (0.93–5.57) | 7 | 1935 | **0.048** | 0.19 |
|  | 3 | 2.34 (0.61–8.63) | 4 | 1266 | 0.19 | 0.77 |
| LAI 1500 m | 1 | 1.79 (0.69–4.05) | 10 | 2201 | 0.19 | 0.38 |
|  | 2 | 1.78 (0.69–4.03) | 10 | 2170 | 0.19 | 0.38 |
|  | 3 | 1.34 (0.40–3.97) | 6 | 1413 | 0.61 | 0.89 |
| NDVI 500 m | 1 | 17.57 (0.03–1.4e+04) | 10 | 2243 | 0.39 | 0.52 |
|  | 2 | 18.87 (0.03–1.3e+04) | 10 | 2210 | 0.37 | 0.50 |
|  | 3 | 0.56 (0.00019–2.1e+03) | 6 | 1431 | 0.89 | 0.89 |
| NDVI 1500 m | 1 | 5.02 (0.0051–5.1e+03) | 10 | 2243 | 0.65 | 0.65 |
|  | 2 | 5.57 (0.0059–5.3e+03) | 10 | 2210 | 0.62 | 0.62 |
|  | 3 | 0.50 (0.0001–3.1e+03) | 6 | 1431 | 0.87 | 0.89 |

Odds ratio (OR) for the association between residential Leaf Area Index (LAI) and Normalized Difference Vegetation Index (NDVI) and risk of celiac disease within 500 m and 1500 m buffers around the child’s home at age 15 years follow-up in the CiPiS cohort. Model 1 = crude estimates. Model 2 = adjusted for sex, maternal age at delivery, season of birth, and maternal smoking during pregnancy. Model 3 = fully adjusted for all available covariates (see Supplemental Table S1). Reported is the p-value of the OR and Benjamini-Hochberg FDR adjustment.
