# Supplementary material for: Associations between residential greenness, land cover and risk of celiac disease in genetically at‐risk children: Celiac Prediction in Skåne study
Source: J Pediatr Gastroenterol Nutr. 2026 Apr 22;83(1):127–34. doi: 10.1002/jpn3.70440 (PMC13342773; doi:10.1002/jpn3.70440)
Supplement: Supplementary file 14 — Supplemental Table S14 (1). [file JPN3-83-127-s012.docx]

| ***Supplemental Table S14*. Sensitivity analysis of the fully adjusted model for the association between Leaf Area Index at 1500m radius and celiac disease at age 9 years screening in the CiPiS study.** | | | | |
| --- | --- | --- | --- | --- |
| **Model** | **N** | **OR (95% CI)** | **p.value** | **p.adj** |
| Full | 1495 | 1.91 (1.20-2.96) | **0.005** | **0.02** |
| Model 2 | 2232 | 1.48 (1.01-2.12) | **0.04** | 0.08 |
| - No Sex | 2232 | 1.50 (1.03-2.15) | **0.03** | 0.06 |
| - No Season of birth | 2232 | 1.50 (1.03-2.15) | **0.03** | 0.06 |
| - No Maternal age | 2232 | 1.47 (1.01-2.11) | **0.04** | 0.08 |
| - No Maternal smoking during pregnancy | 2239 | 1.48 (1.02-2.13) | **0.04** | 0.07 |
| - No Mother working away from home during pregnancy | 2239 | 1.47 (1.01-2.11) | **0.04** | 0.08 |
| - No Maternal educational level | 2236 | 1.49 (1.03-2.14) | **0.03** | 0.06 |
| - No Mother born in Sweden | 2258 | 1.44 (0.99-2.07) | 0.05 | 0.10 |
| - No Paternal educational level | 2244 | 1.58 (1.09-2.25) | **0.01** | **0.03** |
| - No Father born in Sweden | 2236 | 1.48 (1.01-2.12) | **0.04** | 0.08 |

Full model represents the covariates sex, season of birth, mother’s age at delivery, mother smoking during and after pregnancy, if mother was working away from home during pregnancy, parental education level, father’s smoking status, if parents were born in Sweden. Sensitivity analyses were performed on *Model 2*, full adjustment except parental smoking status, by excluding one covariate at a time. Odds ratios (OR) and 95% confidence intervals (CI) were estimated using logistic regression. Adjusted p-values are based on Benjamini-Hochberg correction within the screening age.
